# Deep Learning-Based Detection Model for Leukemia Cells in Peripheral Blood Smears Using YOLOv11-Large

**DOI:** 10.3390/curroncol33080486

**Published:** 2026-08-18

**Authors:** Johan M. Diaz, Arunima Deb, Alexandra Lyubimova, Cedric Nasnas, Leily Santos, Carla Romagnoli, Jacqueline C. Barrientos

**Affiliations:** 1 Department of Internal Medicine, Mount Sinai Medical Center of Florida, Miami Beach, FL 33140, USA; 2 Department of Pathology, Mount Sinai Medical Center of Florida, Miami Beach, FL 33140, USA; 3 Department of Hematology/Oncology, Mount Sinai Comprehensive Cancer Center, Miami Beach, FL 33140, USA

**Keywords:** leukemia, white blood cells, object detection, YOLOv11, peripheral blood smear, deep learning, hematology

## Abstract

Background: Accurate identification and classification of white blood cell (WBC) subtypes in peripheral blood smears (PBS) is essential for the diagnosis and monitoring of hematological malignancies, including leukemia. Conventional manual microscopy, although clinically established, is labor-intensive and subject to inter- and intra-observer variability. Deep learning-based object detection offers a route to automation, yet most prior studies are limited by small datasets, restricted cell taxonomies, or single-microscope acquisition. This study evaluates a YOLOv11-large (YOLOv11L) detector for simultaneous localization and classification of 13 leukemia-relevant WBC subtypes plus an artifact class (14 classes total), trained on the large-scale, multi-domain, open-source LeukemiaAttri dataset. Methods: From the LeukemiaAttri dataset, 18,664 annotated images (67,347 objects) acquired at 40× and 100× magnification were partitioned by stratified sampling into training (70%), validation (15%), and test (15%) sets. The training set was expanded to 65,785 images through extensive geometric, photometric, and AugMix augmentation. A YOLOv11L model pretrained on MS COCO was fine-tuned for 250 epochs (640 × 640 input) on a single NVIDIA H200 SXM GPU, using an auto-selected optimizer (momentum 0.9; weight decay 5 × 10^−4^), automatic mixed precision (AMP), and mosaic augmentation for the first 240 epochs. Results: On an internal held-out test set, the model achieved an mAP50 of 93.9%, mAP50-95 of 77.9%, precision of 94.1%, recall of 88.8%, and an F1 score of 0.913, with similar performance in the validation and test sets. Class-wise average precision (AP) ranged from 89.3% (monocyte) to 98.2% (monoblast), confirming consistent detection across morphologically diverse subtypes. Conclusions: The YOLOv11L detector achieved high performance across all 14 categories on the internal test set, with metrics exceeding those previously reported for subset-specific baselines. These findings support further evaluation of the model as a decision-support tool for peripheral blood smear analysis. External validation is required to determine its clinical utility and generalizability.

## Simple Summary

Leukemia is a cancer in which abnormal white blood cells accumulate in the body. Its initial evaluation commonly includes examination of stained blood films by specialists who identify and classify different cell types under a microscope. This process requires considerable expertise and may vary among observers. We evaluated an artificial intelligence model that locates and classifies cells in blood-film images. The model was trained using a large public dataset containing images acquired at 40× and 100× magnification with both high- and low-cost microscopes and cameras. It achieved high average precision across 14 cell and artifact categories on an internal image-level test set. These results support further evaluation of automated cell detection as an aid for blood-film review.

## 1. Introduction

### Background

Leukemia is a hematologic malignancy characterized by the uncontrolled proliferation of abnormal white blood cells (WBC) arising from genetic alterations in hematopoietic precursor cells. It accounts for approximately 2.4% of global cancer incidence, with an estimated 486,777 new cases annually, and remains a leading cause of cancer-related mortality among young individuals [1,2]. The initial evaluation of suspected leukemia relies on microscopic examination of peripheral blood smears (PBS). In routine clinical practice, trained hematologists or medical technologists assess stained blood films to identify, enumerate, and characterize white blood cell subtypes based on morphological features [2]. These morphological findings inform subsequent diagnostic evaluation, including bone marrow examination and flow cytometry immunophenotyping [3].

However, manual analysis is associated with well-documented limitations. First, the process is inherently labor-intensive, as cell differentiation requires careful examination of multiple microscopic fields at high magnification [2]. Inter-observer and intra-observer variability also represent a persistent concern, particularly when distinguishing morphologically similar cell types [4]. Likewise, access to specialized expertise and advanced diagnostic infrastructure is unevenly distributed; resource-constrained settings often lack both the necessary laboratory equipment and trained hematology professionals required for accurate diagnosis [2,5]. These limitations contribute to diagnostic delays and potential misclassification, which may adversely impact clinical outcomes [5].

Efforts to automate blood cell analysis have a long history dating back to the pioneering work of Wallace Coulter in the mid-twentieth century, which established the foundation for electronic cell counting [6]. Under the Coulter principle, cells suspended in a conductive electrolyte pass through a narrow aperture across which an electrical current is maintained. As each cell passes through the aperture, it displaces an equivalent volume of electrolyte, producing a transient change in impedance proportional to cell volume. This allows cells to be counted and sized simultaneously. Over the past decade, deep learning has driven substantial advances in medical image analysis, with convolutional neural networks (CNNs) achieving performance comparable to clinical experts in tasks such as dermatologic lesion classification and retinal disease detection [7,8]. In hematology, deep learning approaches have been widely applied to WBC classification, segmentation, and detection, leveraging diverse model architectures, including real-time object detection frameworks from the YOLO (You Only Look Once) family [9,10,11].

Architecturally, YOLO detectors comprise three components. A convolutional backbone extracts hierarchical features at progressively coarser spatial resolution; a neck fuses features across scales, typically through a feature pyramid combined with path aggregation, so that both small and large objects are represented; and a detection head emits dense predictions across the resulting feature maps [11]. Single-stage detector models, exemplified by the YOLO family, directly predict an object’s bounding box and class probabilities in a single pass, enabling real-time inference while maintaining competitive accuracy [12]. Unlike two-stage detectors such as Faster R-CNN, which first propose candidate regions and then classify them, YOLO predicts box coordinates and class scores for all locations in a single forward pass, followed by non-maximum suppression to remove duplicates. Recent developments have further improved performance by incorporating attention mechanisms, enhanced feature extraction strategies, and multi-scale detection architectures [13].

Despite these advances, applying deep learning to WBC detection in leukemia diagnosis remains challenging. Many commonly used benchmarks rely on relatively small datasets, include only a subset of WBC types, are acquired under uniform imaging conditions, or lack bounding-box annotations required for object detection tasks [2]. Rehman et al. introduced the LeukemiaAttri dataset to address these limitations. It comprises 28,992 images captured using two microscopes at different cost tiers and three magnification levels, with multiple camera systems [2]. At the highest magnification (100×), experienced hematologists annotated around 10,300 WBC instances and artifacts [2]. It is a richly annotated, multi-domain leukemia dataset, providing a foundation for training and evaluating object detection models under realistic conditions.

Several studies have demonstrated the effectiveness of YOLO-based models for blood cell detection. Jiang et al. proposed an attention-guided YOLOv3 framework that incorporates attention mechanisms, achieving an approximate 7% improvement in mean average precision (mAP) for blood cell detection [14]. In object detection, localization accuracy is commonly assessed using Intersection over Union (IoU), which quantifies the ratio of overlap between a predicted bounding box and the ground-truth bounding box relative to their combined area. Mean average precision at 50% (mAP50) is a metric that evaluates both classification and localization, considering a detection correct if the IoU is 50% or higher. Guo and Zhang further improved YOLOv5 by integrating attention modules and refining the bounding-box calculations, achieving an mAP50 of 97.4% on blood cell datasets [15]. Later, Rehman et al. introduced the AttriDet model together with the LeukemiaAttri dataset. The AttriDet model is a detector built on the YOLOv5 architecture that adds a parallel attribute-prediction branch to the standard detection head, enabling simultaneous prediction of WBC type and associated morphological features [2]. It demonstrated improved performance over baseline YOLOv5 on the LeukemiaAttri dataset [2].

More recently, Abou Ali et al. conducted a comprehensive benchmarking study of the five YOLOv11 variants (nano through xlarge) on a curated 12-class peripheral blood cell dataset comprising 16,891 images and 298,850 annotated cells [16]. Their results demonstrated that the YOLOv11-medium variant achieved the most favorable accuracy–efficiency balance among the evaluated variants, reaching an mAP50 of 93.4%, while larger models provided only marginal performance gains at substantially higher computational cost [16]. Furthermore, a dataset split into training (80%), validation (10%), and test (10%) sets (80:10:10 split) consistently outperformed a 70:20:10 split configuration across all model variants, suggesting that increased training data volume improves model generalization in cytological detection tasks [16]. However, their evaluation was conducted on images acquired from a single automated analyzer and limited to normal peripheral blood cell types, leaving uncertainty regarding the generalizability of YOLOv11 to multi-domain datasets and pathological conditions such as leukemia.

To address these gaps, this study makes three key contributions. First, we leverage the LeukemiaAttri dataset, a large-scale, publicly available, multi-domain dataset that provides bounding-box annotations for diverse WBC subtypes derived from patients with leukemia, supporting evaluation across several acquisition configurations. Second, we evaluate a YOLOv11-large (YOLOv11L) object detection model using an open-source YOLO implementation, which incorporates recent architectural advances to enable simultaneous localization and classification of 13 WBC categories and one artifact category, for a total of 14 classes. Third, we describe the training and evaluation procedure that incorporates extensive data augmentation, and report comprehensive performance metrics, including precision, recall, F1 score, and mean average precision metrics on both the validation and internal test sets.

## 2. Materials and Methods

### 2.1. Dataset

This study used the LeukemiaAttri dataset, a large-scale, multi-domain benchmark for WBC detection enriched with morphological attribute annotations [2]. The dataset was collected from peripheral blood films of leukemia patients at a diagnostic laboratory in Lahore, Pakistan, with appropriate patient consent. Images were acquired using two microscopes spanning different cost tiers: a high-cost Olympus CX23 (Olympus Corporation, Tokyo, Japan; HCM) and a low-cost XSZ-107BN (Ningbo, China; LCM). For each microscope, data were captured at three magnification levels (10×, 40×, and 100×) using multiple imaging devices, including an HD1500T high-end camera (Meiji Techno Co., Ltd., Saitama, Japan; HCM), a ZZCAT 5MP camera (Zhuzhou Carter Photoelectric Instrument Co., Ltd., Zhuzhou, China; LCM), and an Honor 9X Lite smartphone camera (Honor Device Co., Ltd., Shenzhen, China) for both systems. This design introduces controlled domain variability across microscopes, magnifications, and acquisition devices, and was used here as a source of training diversity. The dataset comprises approximately 28,992 images organized into multiple domain-specific subsets, with over 10,300 annotated WBC instances and artifacts. All reference annotations reflect expert morphological classification of stained smears. Individual cells were not confirmed by immunophenotyping, immunohistochemistry, or molecular studies, so the labels represent morphological category rather than immunophenotypically verified lineage.

Expert hematologists manually annotated approximately 10,300 WBC instances acquired at 100× magnification on the high-cost microscope with the HD1500T camera [2]. Because a stained blood film is an essentially planar specimen imaged along a fixed optical axis, the geometric relationship between two images of the same field acquired under different magnifications or with different cameras is well approximated by a planar projective transformation, or homography, which is a 3 × 3 matrix mapping points in one image to the corresponding points in the other. Rehman et al. estimated this transformation for each field from corresponding image features, applied it to the coordinates of each reference bounding box, and re-fitted axis-aligned boxes to the transformed corners, thereby transferring annotations from the reference configuration to the remaining magnifications and imaging devices [2]. Each propagated annotation was then manually verified and refined by the dataset authors. The dataset includes 14 WBC classes spanning both myeloid and lymphoid lineages, including a “none” (artifact) class [2].

For this study, we combined images acquired at 40× and 100× magnifications while excluding 10× images. The 10× subset was omitted due to its lower spatial resolution and increased prevalence of blurring artifacts, which limit the visibility of fine cytomorphologic features and may adversely impact model performance. This approach aligns with standard hematopathology workflows, in which definitive leukocyte characterization is performed at higher magnification. The inclusion of both 40× and 100× images provides wider field context at 40× and greater cellular detail at 100×. Following preprocessing, corrupted files and images with missing annotations were removed, yielding a final dataset of 18,664 images and 67,347 annotated objects (Table 1). The 14 classes included were abnormal promyelocyte, atypical lymphocyte, basophil, eosinophil, lymphoblast, lymphocyte, metamyelocyte, monoblast, myeloblast, myelocyte, neutrophil, promonocyte, monocyte, and “none” (artifact). The annotation files did not contain a separate promyelocyte class; abnormal promyelocyte was retained among the 13 WBC categories, together with the “none” artifact category, for a total of 14 classes. The class distribution was substantially imbalanced, with most annotations concentrated in the “none” (artifact), myeloblast, lymphoblast, and neutrophil categories, whereas rare classes such as basophil, eosinophil, and metamyelocyte were sparsely represented (Table 1). Class imbalance across WBC subtypes was mitigated during training using preferential data augmentation.

### 2.2. Data Splitting and Augmentation

The 18,664 images were partitioned into training (70%), validation (15%), and test (15%) sets using stratified random sampling at the image level to preserve class distribution across all splits. This splitting strategy was used following recent benchmarking studies of YOLO architectures that emphasize the importance of maintaining representative validation and test subsets for reliable performance estimation. The internal test set was strictly held out from all training and model selection procedures. Randomization was performed at the image level. Patient-level and field-level identifiers are not distributed with the LeukemiaAttri dataset, so grouped splitting was not possible. Given that annotations in this dataset were propagated across magnifications, cameras, and microscopes, images of the same physical cell acquired under different configurations are expected to be distributed across splits. Because dataset partitioning was performed at the image level rather than the object level, the resulting per-class annotation counts did not exactly follow the nominal 70:15:15 split.

To address class imbalance and enhance model generalization, augmentation was applied exclusively to the training set, expanding the training set to a total of 65,785 images. A custom Python 3.10 script applied image augmentations based on class labels. Augmentation was applied preferentially to images containing underrepresented classes rather than distributed evenly across the training set. The augmentation process was designed to simulate realistic variations in microscopy imaging while preserving underlying cellular morphology. Geometric transformations included horizontal and vertical flipping, fixed 90° rotations, random rotations within ±15°, and shear transformations up to ±15° along both axes. Photometric augmentations included brightness adjustment (±25%), saturation shift (±30%), and exposure adjustment (±15%) to account for differences in staining intensity and illumination. Additional perturbations included a Gaussian blur of up to 3 pixels and stochastic noise injection affecting up to 1.96% of pixels, simulating sensor noise and acquisition artifacts. AugMix was also applied in training to enhance robustness through stochastic combinations of augmentations and blended outputs, thereby improving resilience to domain shift without changing semantic labels. This augmentation strategy was particularly important given the multi-domain structure of the LeukemiaAttri dataset, which includes substantial variability across microscopes, magnifications, and imaging devices.

All augmentations were selected to preserve the diagnostic identity of the cell. Rotation and flipping reflect the arbitrary orientation of cells on a smear, and photometric adjustments reflect genuine variation in staining and illumination across laboratories. Shear transformation is the least conservative of the transformations applied, since it alters cell aspect ratio and may perturb nuclear contour and apparent nuclear-to-cytoplasmic ratio, and it was accordingly restricted to ±15%. Augmentation was applied exclusively to the training split, so reported performance reflects evaluation on unmodified images.

### 2.3. Model and Training

We employed the YOLOv11L object detector, an object detection model designed for efficient and high-resolution feature learning. The model consists of a CSP-based backbone for hierarchical feature extraction, a feature pyramid and path aggregation neck for multi-scale feature fusion, and an anchor-free detection head that simultaneously predicts bounding boxes, objectness, and class probabilities. The YOLOv11L variant (approximately 25.3 million parameters) provides a balance between computational efficiency and representational capacity, enabling discrimination of subtle cytomorphologic differences between closely related leukocyte subtypes. A single model was trained on the pooled 40× and 100× images. Separate magnification-specific models were not trained, and reported metrics are pooled across magnifications and across microscope tiers. The YOLOv11L variant was selected a priori based on published benchmarking of YOLOv11 variants for peripheral blood cell detection [16]. No internal comparison against other variants or architectures was performed.

The model was initialized with weights pretrained on the MS COCO image dataset to leverage transfer learning and accelerate convergence on this specialized hematologic imaging task. Training and evaluation were performed on a cloud instance with a single NVIDIA H200 SXM GPU (141 GB VRAM), 12 virtual CPU cores, and 188 GB system RAM, running Ubuntu Linux, requiring approximately 23.5 h end-to-end. The software environment comprised Python 3.10, PyTorch 2.4, CUDA 12.4, and Ultralytics 8.3.170. Images were resized to 640 × 640 by stretching, applied identically to all splits. Random seeds were not fixed. The training environment was ephemeral and is no longer available, and training scripts and console logs were not retained. Hyperparameter selection was explored by random search over learning rate and batch size, evaluated on the validation split; no sampled configuration improved on the settings produced by the framework’s automatic optimizer selection, which was therefore adopted for the final model. Under the automatic mode of the Ultralytics framework, the optimizer, initial learning rate, and momentum are selected according to the total number of optimization iterations. For the present configuration this resolved to stochastic gradient descent with an initial learning rate of 0.01 and momentum of 0.9, with a weight decay of 5 × 10^−4^. A cosine annealing learning-rate schedule was enabled, and batch size was determined automatically from available GPU memory. Automatic mixed precision (AMP) was enabled to accelerate training and reduce memory usage.

Training used mosaic augmentation in addition to the pre-generated augmented images. Mosaic augmentation was used for the first 240 epochs, enabling the model to learn from diverse object scales and contextual compositions by combining multiple images into a single training sample. This was disabled in the final 10 epochs to allow fine-tuning on natural image distributions and improve localization precision. AugMix augmentation was applied during training to enhance robustness to domain shift by introducing stochastic combinations of photometric and geometric transformations. All images were resized to an input resolution of 640 × 640 pixels. This resolution was selected for compatibility with the pretrained model configuration and the available resources.

Annotations were stored in YOLO format: one plain-text file per image, each line encoding a class index and box center coordinates, width, and height normalized to image dimensions. Model outputs were written to the framework’s standard run directory, comprising the trained weights, a per-epoch metrics log, and generated figures. Test-set predictions were exported as per-image text files containing class index, box coordinates, and confidence scores. No patient-identifiable data were stored at any stage.

### 2.4. Evaluation Metrics

Model performance was evaluated using standard object detection metrics, including precision, recall, F1 score, and mean average precision (mAP). Precision was defined as the proportion of predicted positive detections that were correct and reflects the model’s reliability and susceptibility to false-positive predictions. Recall, or sensitivity, was defined as the proportion of true positive instances that were correctly detected. The F1 score, calculated as the harmonic mean of precision and recall, was used as a summary measure of overall detection performance, particularly in settings with class imbalance.

mAP was selected as the primary detection metric because it incorporates both classification performance and localization accuracy. We report mAP50, defined at an Intersection over Union (IoU) threshold of 0.50, and mAP50-95, which averages performance across IoU thresholds from 0.50 to 0.95 in increments of 0.05. Whereas mAP50 reflects detection performance under a more permissive localization criterion, mAP50-95 provides a more stringent assessment by penalizing imprecise bounding box placement. All metrics were calculated on the validation set for performance monitoring during training and on the held-out test set for final evaluation.

## 3. Results

### 3.1. Overall Detection Performance

**Figure 1:**
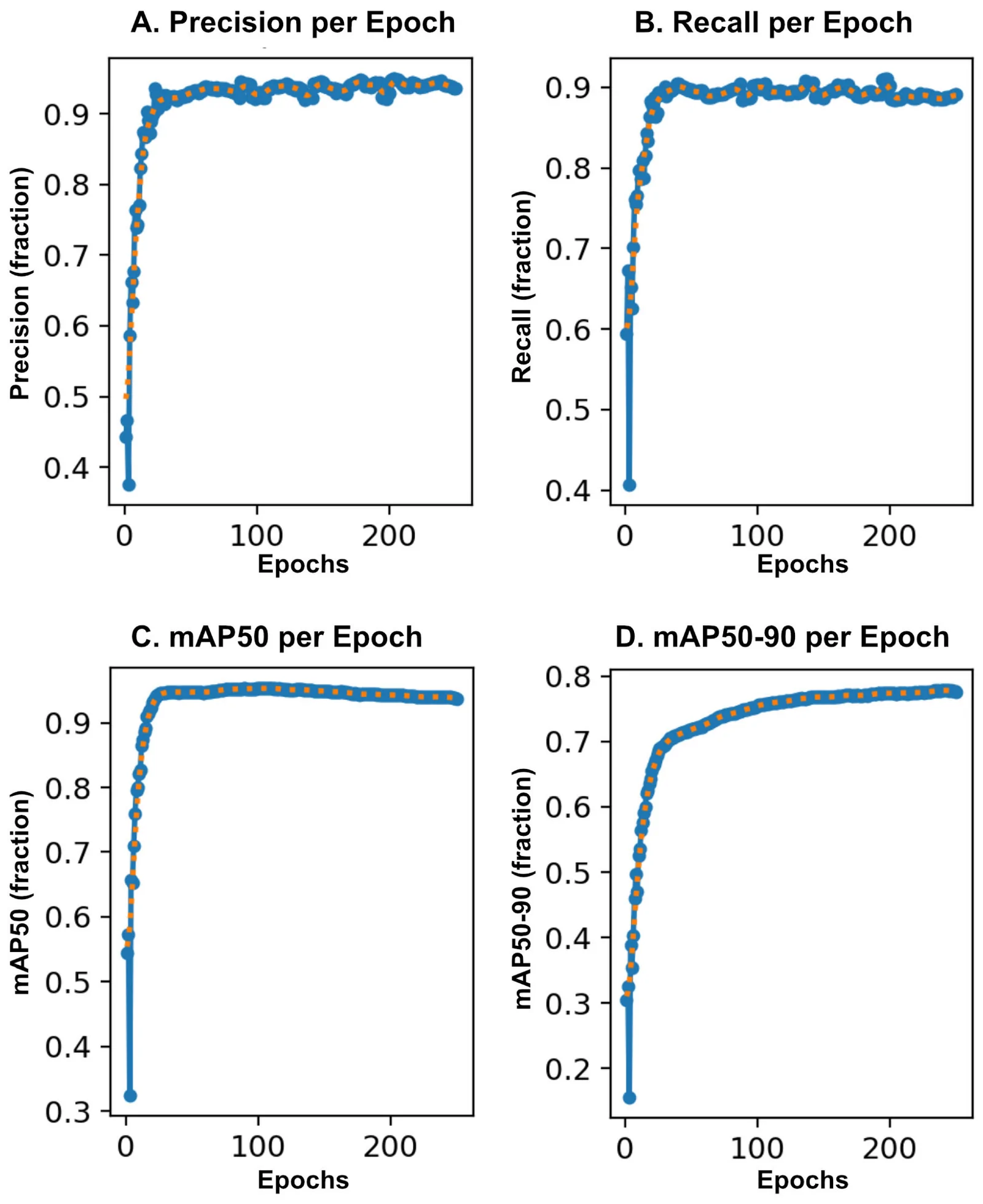
Model performance metrics across training epochs. The curves illustrate the evolution of precision, recall, mean average precision at IoU 0.5 (mAP50), and mean average precision averaged over IoU 0.5–0.95 (mAP50-95) during training and validation. Each metric is plotted against training epochs, demonstrating progressive model improvement and stabilization. Values are reported as fractions. Reprinted with permission from ref. [17]. *© American Society of Hematology 2025. Reused with permission*.

The YOLOv11L model demonstrated strong, consistent performance throughout training, with metrics stabilizing in later epochs, as shown in Figure 1. At the end of 250 training epochs, the model achieved a validation precision of 93.6% and recall of 89.2%, yielding an mAP50 of 93.8% and mAP50-95 of 77.6% (Table 2; Figure 1). On the internal held-out test set, the model achieved mAP50 of 93.9% and mAP50-95 of 77.9%, with precision of 94.1% and recall of 88.8% (Table 2). The minimal difference between validation and test performance (mAP50 of 93.8% vs. 93.9%) suggests stable optimization. Performance across these splits does not exclude correlation between related images. The model evaluated on the test set achieved an F1 score of 0.913, with precision and recall of 94.1% and 88.8%, respectively.

To assess the model’s ability to detect individual WBC subtypes, we examined class-wise average precision (AP) values (Table 3). Performance remained consistently high across all classes, with AP values ranging from 89.3% to 98.2%. The highest detection performance was observed for monoblasts (98.2%) and atypical lymphocytes (98.1%), followed by neutrophils (96.8%), abnormal promyelocytes (96.7%), myeloblasts (96.1%), and lymphoblasts (95.9%). Performance also remained strong for eosinophils (93.5%), myelocytes (93.1%), lymphocytes (93.0%), and promonocytes (92.9%), while somewhat lower values were observed for the “none” (artifact) category (92.5%), basophils (89.7%), metamyelocytes (89.5%), and monocytes (89.3%). The relatively lower performance in some subclasses may reflect overlapping morphological features among closely related cells, particularly within immature myeloid and monocytic populations, where similarities in nuclear contour, chromatin pattern, and cytoplasmic appearance can make discrimination more challenging. Nonetheless, AP exceeded 89% for every class in the internal test set.

**Figure 2:**
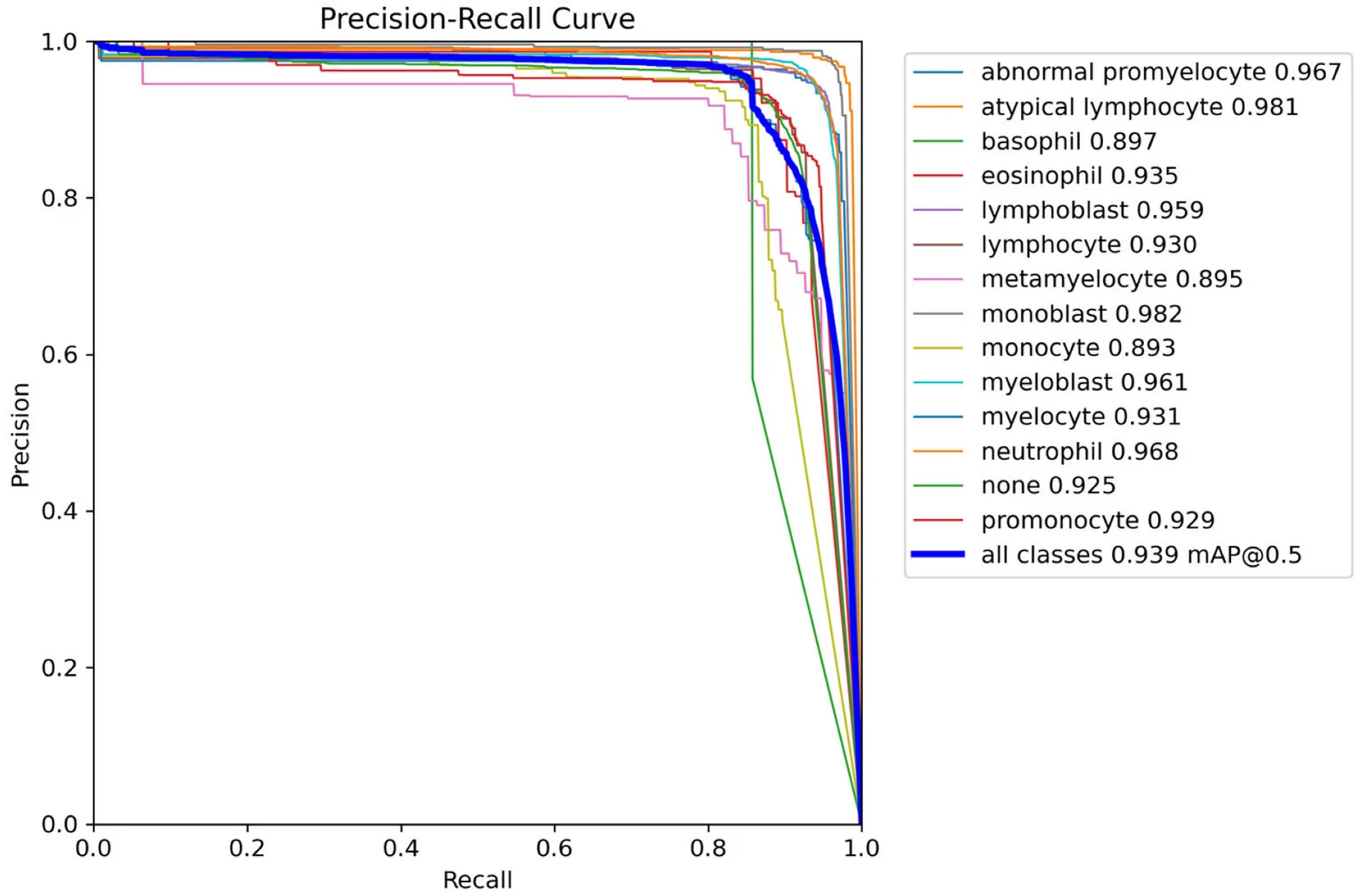
Precision–recall curve for all 14 classes on the held-out test set. The bold blue curve denotes the mean across classes (mAP50 = 0.939); thin curves denote individual classes, with per-class AP given in the legend as a fraction.

The model’s precision–recall characteristics are summarized in Figure 2. The aggregate area under the curve corresponded to an mAP50 of 0.939, with precision remaining above 0.95 across most of the recall range before declining sharply as recall approached 1.0. The narrow spread of the per-class curves indicates that detection quality was maintained across both abundant and rare subtypes.

**Figure 3:**
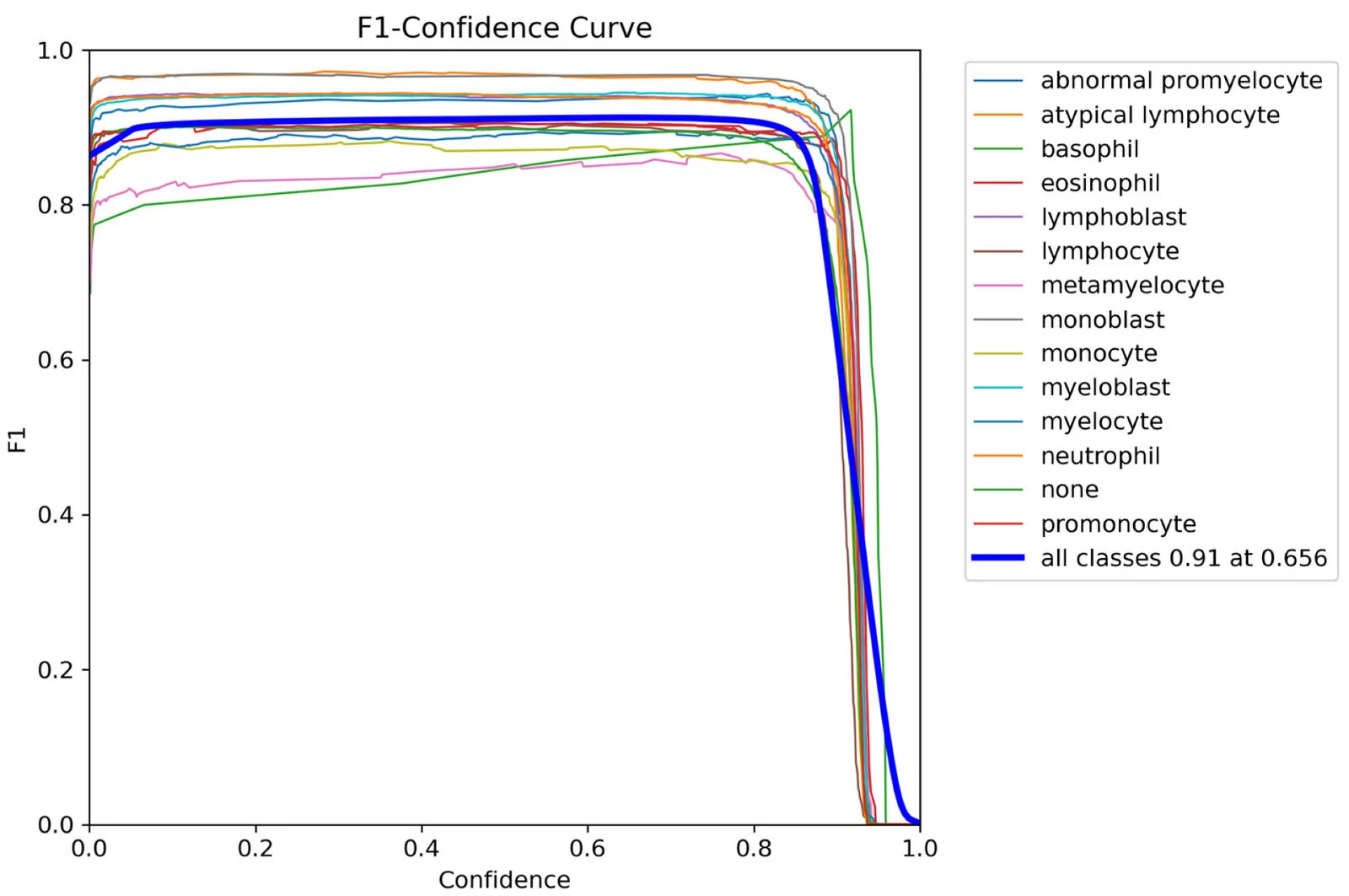
F1–confidence curve for all 14 classes on the held-out test set. The bold curve denotes the mean across classes, which peaks at F1 = 0.91 at a confidence of 0.656; thin curves denote individual classes.

The F1–confidence relationship is shown in Figure 3. Aggregated across all classes, F1 peaked at 0.91 at a confidence threshold of 0.656, in close agreement with the calculated test-set F1 of 0.913 reported above based on precision and recall values for the test set. The curve remained high and approximately flat across a broad confidence range (roughly 0.05–0.85) before falling steeply at very high thresholds, indicating balanced performance and did not depend on narrow threshold tuning.

**Figure 4:**
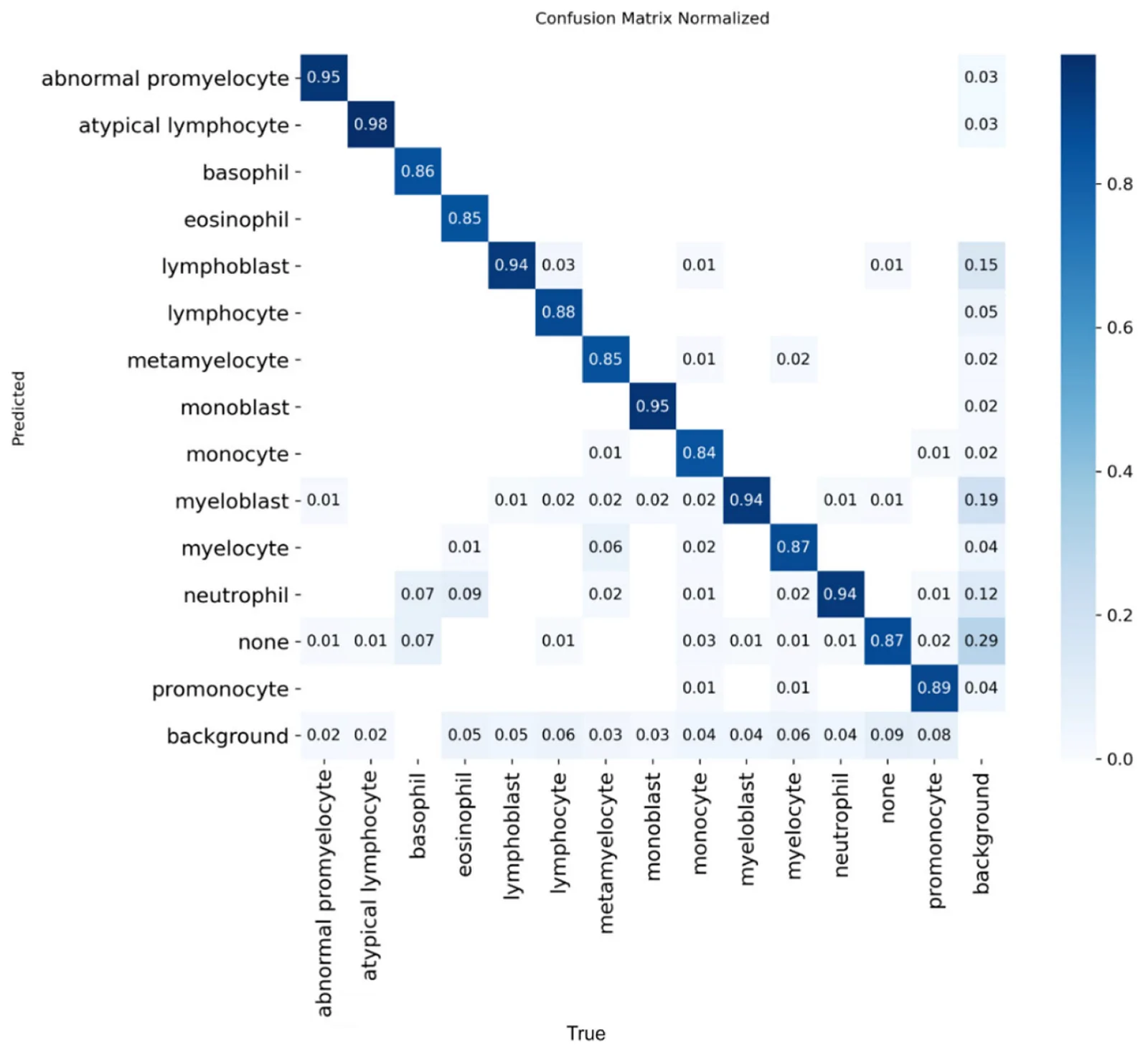
Normalized confusion matrix on the held-out test set. Columns correspond to ground-truth classes and rows to predicted classes; values are normalized per ground-truth column, so diagonal entries give per-class recall. The background row and column capture missed detections (true cells predicted as background) and false positives (background predicted as a cell), respectively.

The normalized confusion matrix (Figure 4, un-normalized data see Figure S1) summarizes class-level prediction behavior, with columns normalized by the number of true instances per class so that diagonal entries represent per-class recall at the operating point. Recall was high for most classes, ranging from 0.84 (monocyte) to 0.98 (atypical lymphocyte). The principal off-diagonal confusions occurred between morphologically related cells: a fraction of true metamyelocytes were predicted as myelocytes (0.06), and true basophils and eosinophils were occasionally predicted as neutrophils (0.07 and 0.09, respectively). The largest source of error involved the background class, encompassing both missed detections (true cells predicted as background, 0.02–0.09 across classes) and false positives (background predicted as a cell, most often as “none” (0.29), myeloblast (0.19), lymphoblast (0.15), and neutrophil (0.12)). These patterns are consistent with the decline in precision at high recall seen in Figure 2 and with the recognized difficulty of separating immature myeloid and monocytic forms.

### 3.2. Qualitative Detection Results

**Figure 5:**
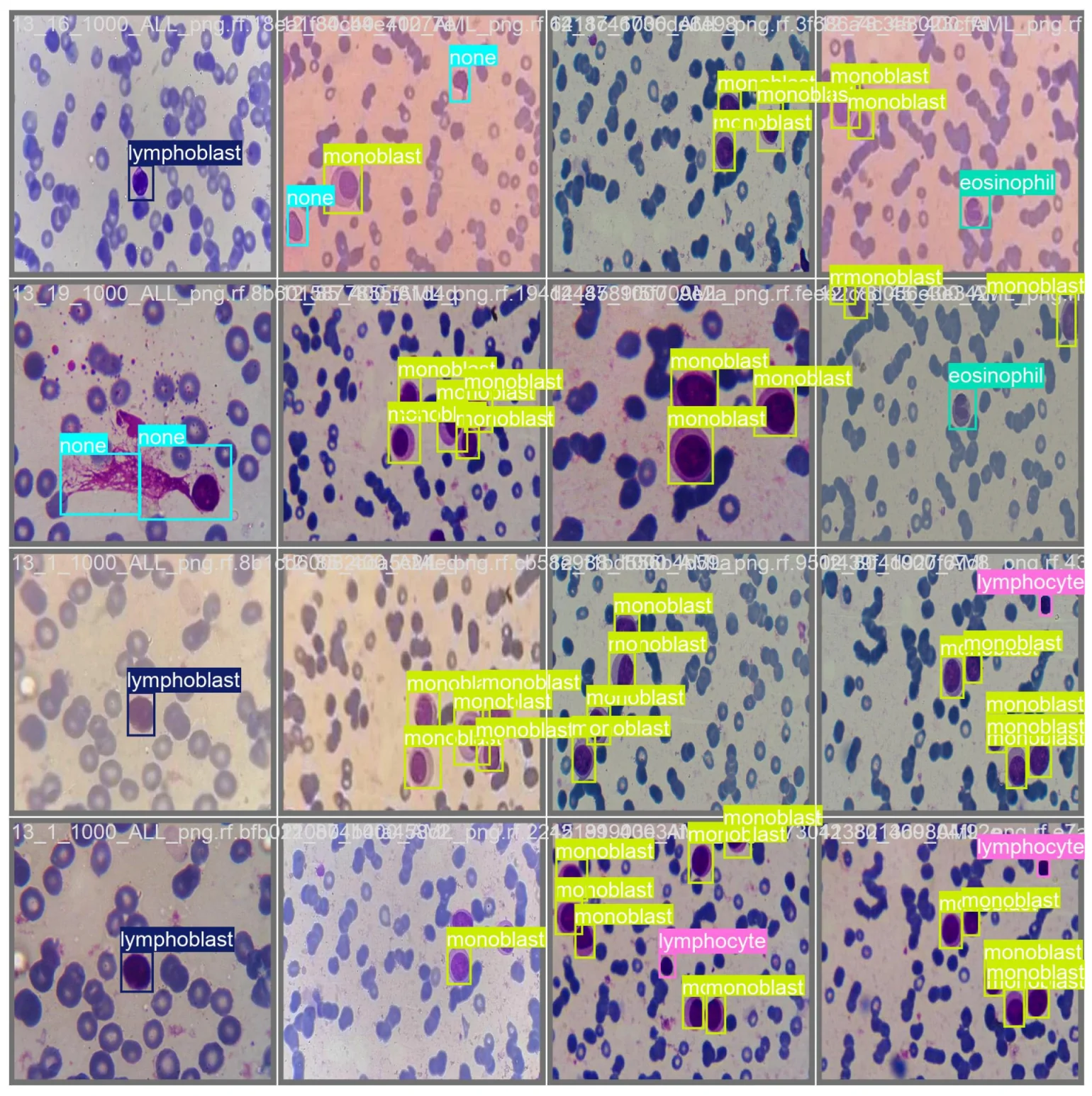
Ground-truth annotations on validation batch images. Each panel shows a peripheral blood smear image with expert-annotated bounding boxes and class labels for WBC subtypes (monoblast, lymphoblast, eosinophil, lymphocyte) and “none” (artifact). The grid illustrates the diversity of imaging conditions, cell densities, and staining characteristics in the LeukemiaAttri dataset. Source images from the LeukemiaAttri dataset [2].

**Figure 6:**
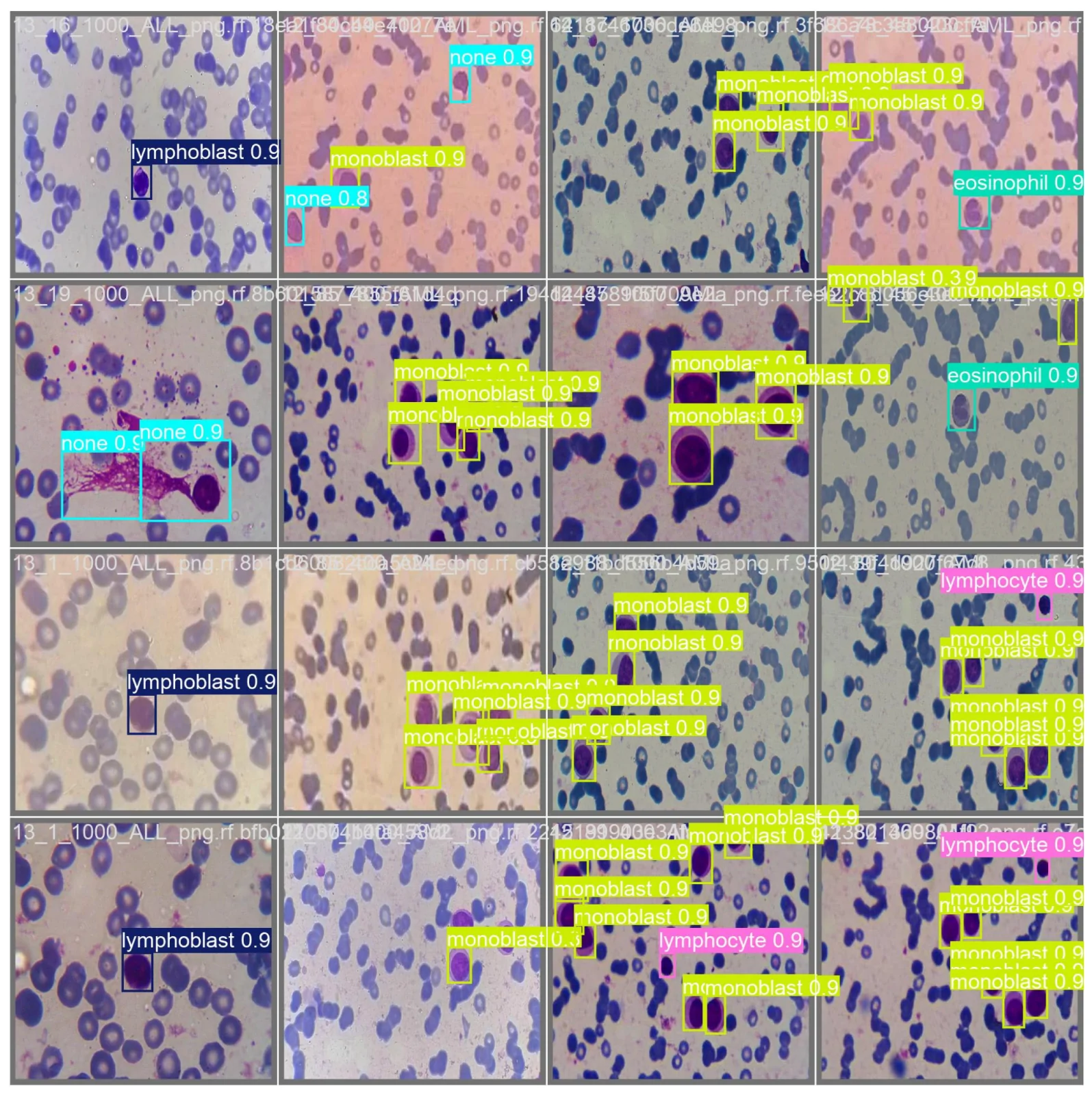
Model predictions on the same validation batch images shown in Figure 5. Predicted bounding boxes with class labels and confidence scores (shown as decimal values) demonstrate close alignment with ground-truth annotations. The majority of detections achieve confidence scores of 0.9 or above, showing close agreement with the displayed reference annotations. Source images from the LeukemiaAttri dataset [2].

To complement the quantitative metrics, Figure 5 and Figure 6 show a qualitative comparison of ground-truth annotations and model predictions on representative validation images. Figure 5 displays the reference bounding boxes and class labels for 16 validation images containing lymphoblasts, monoblasts, eosinophils, lymphocytes, and “none” (artifact) regions. These examples highlight the substantial visual heterogeneity of the LeukemiaAttri dataset, including variation in staining, background intensity, cell density, and image quality.

Figure 6 shows the corresponding model predictions for the same images. Predicted boxes closely matched the reference annotations in both position and extent, indicating accurate localization and classification. Confidence scores were generally high, with most detections at or above 0.9. The model also performed well in crowded fields containing multiple closely adjacent cells, where it was able to separate individual monoblasts with distinct bounding boxes. Overall, the qualitative agreement between annotations and predictions supports the quantitative results and indicates that model performance was maintained across visually diverse validation samples.

### 3.3. Computational Performance

The YOLOv11L model contains 25.3 million parameters, with a reported complexity of approximately 86.9 GFLOPs at 640 × 640 input [11]. To assess feasibility on hardware representative of resource-constrained deployment, the trained model was served through a browser-based application on a CPU-only virtual server with 2 vCPUs and 4 GB RAM, without GPU acceleration. Mean end-to-end inference time in this configuration was 2312.5 ms per image, corresponding to a throughput of approximately 0.43 images per second, or roughly four minutes for a 100-field scan. Inference latency on the training-time hardware was not benchmarked.

## 4. Discussion

### 4.1. Comparison with Prior Work

Our YOLOv11L model achieved higher mAP than the baselines reported by Rehman et al. on LeukemiaAttri subsets [2]. In their original benchmarking, YOLOv5x achieved the highest reported mAP50 of 44.2% and mAP50-95 of 26.3% on the 100× (H_100x_C2) subset [2]. Although direct comparison is limited by differences in subset composition, magnification selection, and training strategy, our model’s mAP50 of 93.9% on the held-out test set was higher than the reported subset-specific baseline values. Several differences may have contributed to the higher metrics. First, we used a substantially larger and more diverse training set by combining the 40× and 100× subsets from both HCM and LCM, whereas prior baselines were evaluated on individual subsets. Similarly, our augmentation pipeline, applied preferentially on the underrepresented classes, expanded the training set from 13,065 images (70% of 18,664 images) to 65,785 images, providing greater training diversity. Lastly, YOLOv11 incorporates architectural advances over YOLOv5, including an anchor-free detection head, enhanced multi-scale feature fusion, and improved loss functions. The AttfriDet model proposed by Rehman et al. could not be directly compared with ours because it emphasizes explainability through a multiheaded WBC detector and was evaluated under different conditions [2]. The contribution of this study lies in its application and evaluation rather than in the development of a new architecture. YOLOv11 was used without structural modification and evaluated on the combined 40× and 100× LeukemiaAttri subsets. To our knowledge, previous LeukemiaAttri studies evaluated individual imaging configurations rather than this broader combination of domains. The model achieved higher detection metrics than previously reported subset-specific baselines. Although direct comparison is limited by differences in datasets and training protocols, these findings suggest that broader data coverage and systematic augmentation may improve performance without requiring architectural modification.

Compared with the YOLOv11 benchmarking study by Abou Ali et al., our mAP50 of 93.9% on the LeukemiaAttri dataset is comparable to their reported mAP50 of 94.7% for the YOLOv11-large variant on the PBC dataset using an 80:10:10 split [16]. We instead used a 70:15:15 split, reserving a larger held-out fraction for validation and testing to obtain more reliable performance estimates on this more challenging dataset while retaining sufficient training data given its scale. The datasets differ in cell taxonomy and acquisition conditions. The LeukemiaAttri dataset contains normal and pathological cell types, and it was captured across multiple microscopes and cameras, introducing significant domain shift. The performance levels suggest that the YOLOv11 architecture, combined with appropriate training strategies, warrants evaluation in external clinical datasets. Abou Ali et al. further observed that performance gains plateau beyond the medium variant, with the large and xlarge models offering only marginal improvements [16]. In our study, the large variant was selected as a compromise between the reported performance and computational requirements of the available variants.

### 4.2. Clinical Implications

After external validation, the model could be studied as a tool for identifying cells that require expert review, particularly in resource-constrained settings. An F1 score of 0.913 indicates a strong balance between precision and recall within this dataset, a useful property for systems intended to flag suspicious cells for expert review. Precision was 94.1% at the selected operating point on the internal test set, whereas the recall of 88.8% indicated that nearly 89% of annotated instances were detected. Whether this error profile is clinically acceptable will depend on the intended use, the consequences of missed cells, and the selected operating threshold. The precision–recall curve (Figure 2) shows that high precision is sustained across most of the recall range, and the F1–confidence curve (Figure 3) confirms that balanced performance remains stable across a wide range of decision thresholds, simplifying the selection of an operating point for future implementation studies.

The class-specific AP values (89.3–98.2%) are encouraging for future clinical evaluation. Monoblasts and atypical lymphocytes, cell types whose presence could suggest specific leukemia subtypes, are consistently detected. Even the most challenging classes (monocytes, metamyelocytes, and basophils) achieve an average precision above 89%, which may be useful for flagging suspicious cells for expert review after validation. The model’s ability to simultaneously localize and classify cells in a single pass enables rapid screening of entire blood films, potentially reducing the time required for manual differential counts. The normalized confusion matrix (Figure 4) further shows that the residual misclassifications fall predominantly between morphologically adjacent cells. Most notably, metamyelocytes are predicted as myelocytes and basophils or eosinophils predicted as neutrophils, rather than between clinically unrelated categories, so that errors tend to remain within the same maturation lineage.

Furthermore, the model’s training on the open, multi-domain LeukemiaAttri dataset, which includes images from both high-cost and low-cost microscopes, suggests potential applicability across different laboratory equipment tiers. This is particularly relevant for resource-limited settings, where access to expensive automated hematology analyzers is limited and where low-cost microscopes paired with mobile phone cameras may serve as the primary imaging modality [2].

Deployment feasibility depends on whether inference is achievable on hardware realistically available in the settings the model is intended to serve. The configuration tested here, a CPU-only server with 2 vCPUs and 4 GB RAM and no dedicated accelerator, completed inference in approximately 2.3 s per field, so a 100-field review would require roughly four minutes. This is not real-time performance at the microscope, but it is compatible with an asynchronous triage workflow in which digitized fields are queued, processed in the background, and surfaced to a hematologist with morphologic flags for confirmation. In routine practice the most plausible integration point is triage: automated pre-screening with flags surfaced in the laboratory information system, mandatory hematologist confirmation of any abnormal finding, and no automated differential released without professional human supervision.

### 4.3. Limitations

Several limitations must be acknowledged. First, while the LeukemiaAttri dataset provides multi-domain variability through different microscopes and cameras, all images originate from a single diagnostic laboratory. External validation using multicenter datasets from geographically diverse populations is essential to assess generalizability. Second, the split was performed at the image level because patient-level and field-level grouping metadata are not available. Given that the dataset contains multiple images of the same cells across acquisition configurations, information leakage between training and test splits is expected rather than merely possible, and the reported metrics are correspondingly optimistic as estimates of performance on new patients. The close agreement between validation and test performance does not mitigate this since both splits are affected equally. Third, the mAP50-95 of 77.9%, while higher than prior baselines on this dataset, indicates room for improvement in precise bounding box localization, particularly for cells that are closely packed or overlapping. In addition, the confusion matrix (Figure 4) shows that the most frequent error mode is confusion between objects and background, comprising both missed detections (up to roughly 9% of cells for some classes) and false-positive detections in background regions (most often labeled as “none”, myeloblast, or lymphoblast); reducing these background errors is an important target for future refinement.

Fourth, the current model does not predict morphological attributes (such as nuclear chromatin pattern or cytoplasmic characteristics), which provide the explainability that hematologists require for confident diagnosis. Future work could integrate attribute-prediction heads similar to the AttriDet approach proposed by Rehman et al. to enhance interpretability [2]. Images were resized to a square input by stretching rather than by preserving their aspect ratios. Consequently, cell morphology was anisotropically scaled in both training and evaluation images. Although applied consistently across all splits, this alters shape features that carry diagnostic information, and aspect-ratio-preserving resizing would be preferable in future work.

Fifth, a CPU-only server with 2 vCPUs and 4 GB RAM completed inference in approximately 2.3 s per field, but inference speed considerations for deployment on edge devices remain to be evaluated. Sixth, performance was not stratified by acquisition configuration. Because metrics are pooled across magnifications, microscopes, and cameras, the possibility that detection performance differs systematically between high-cost and low-cost microscope images is not excluded by these results. This limits the inference that the model is applicable across equipment tiers: that expectation rests on the composition of the training data rather than on stratified evidence.

Seventh, class imbalance was mitigated but not resolved. Preferential augmentation increased the representation of underrepresented classes during training, but augmentation generates additional views of existing cells rather than new cells, so the morphological diversity available for classes such as basophil and metamyelocyte remains limited by the small number of distinct source instances. Finally, as noted by Abou Ali et al., the morphological similarity among immature granulocytes remains a fundamental challenge that may require additional architectural innovations, such as fine-grained attention mechanisms or specialized loss functions, to be fully addressed [16].

### 4.4. Future Directions

The next step is external validation to evaluate the model’s robustness across different patient populations, staining protocols, and imaging equipment. Likewise, incorporating morphological attribute prediction through multi-task learning architectures would enhance the clinical interpretability of the model’s outputs, providing hematologists with structured information about the cells that support leukemia subtype classification. Explainable AI methods such as Grad-CAM or attention visualization could improve the interpretability of individual detections by highlighting the morphological features driving each detection. Finally, integration into existing hematology information systems and prospective clinical trials evaluating the model’s impact on diagnostic accuracy, turnaround time, and workflow efficiency would be required before clinical implementation.

## 5. Conclusions

This study demonstrates that a YOLOv11-large model, trained on the open, multi-domain LeukemiaAttri dataset with extensive augmentation, achieved high internal test-set performance for simultaneous localization and classification of 13 white blood cell subtypes and an artifact class relevant to leukemia diagnosis. On the internal test set, the model achieved an mAP50 of 93.9%, mAP50-95 of 77.9%, and an F1 score of 0.913, representing an improvement over previously published LeukemiaAttri subset baselines while acknowledging differences in subset composition and training strategy. Class-wise average precision ranged from 89.3% to 98.2%, confirming consistent detection across morphologically diverse cell types, including difficult-to-detect immature leukocyte variants. Qualitative inspection of selected validation images showed close alignment between model outputs and expert ground-truth annotations.

The current evidence is limited to the internal image-level split based on the LeukemiaAttri dataset and does not establish clinical performance. However, the results highlight the capability of modern YOLO architectures, combined with appropriate training strategies, to provide potentially useful automated decision support for complex peripheral blood smear analysis. The documented training protocol, consistency across cell types, and efficient single-pass inference support its further evaluation as a candidate decision-support tool in hematology workflows.

Future evaluation should use external patient-level cohorts and report diagnostic accuracy, inference time, and performance across imaging systems. Clinical workflow studies would then be needed to determine whether the detector improves review efficiency without increasing missed clinically important cells. Further research should also explore multi-task architectures incorporating morphological attribute prediction to enhance diagnostic explainability. Ultimately, these findings support further evaluation for the integration of deep learning-based detection into hematological diagnostic processes.

## Figures and Tables

**Table 1:** Per-class annotation counts and proportions across the study subset of 18,664 images, prior to splitting and augmentation.

Class	Objects	Distribution
**None (artifact)**	15,902	23.61%
**Myeloblast**	13,910	20.65%
**Lymphoblast**	11,595	17.22%
**Neutrophil**	8828	13.11%
**Monoblast**	3189	4.74%
**Atypical lymphocyte**	2757	4.09%
**Promonocyte**	2260	3.36%
**Lymphocyte**	2118	3.14%
**Myelocyte**	2099	3.12%
**Abnormal promyelocyte**	1890	2.81%
**Monocyte**	1423	2.11%
**Metamyelocyte**	666	0.99%
**Eosinophil**	597	0.89%
**Basophil**	113	0.17%
**Total**	67,347	100%

**Table 2:** Model performance metrics on validation and test datasets. Precision, recall, and mean average precision (mAP) metrics are reported for both validation and internal test sets. Reprinted with permission from ref. [17]. © *American Society of Hematology 2025. Reused with permission*.

	Validation (%)	Test (%)
Precision	93.6%	94.1%
Recall	89.2%	88.8%
mAP50	93.8%	93.9%
mAP50-95	77.6%	77.9%

**Table 3:** Per-class average precision (AP) and mAP at IoU 0.5 (mAP50) for all classes. This table presents the average precision values for individual leukocyte classes and mAP50 for all classes obtained during model evaluation. Reprinted with permission from ref. [17]. *© American Society of Hematology 2025. Reused with permission*.

Class	AP (%)
Monoblast	98.2%
Atypical Lymphocyte	98.1%
Neutrophil	96.8%
Abnormal Promyelocyte	96.7%
Myeloblast	96.1%
Lymphoblast	95.9%
Eosinophil	93.5%
Myelocyte	93.1%
Lymphocyte	93.0%
Promonocyte	92.9%
None (artifact)	92.5%
Basophil	89.7%
Metamyelocyte	89.5%
Monocyte	89.3%
All Classes	93.9%

## Data Availability

The dataset analyzed in this study, the LeukemiaAttri dataset, is publicly available and was not generated by the present authors. It was originally introduced by Rehman et al. [2] and can be accessed through the project repository at https://github.com/intelligentMachines-ITU/Blood-Cancer-Dataset-Lukemia-Attri-MICCAI-2024, (accessed on the 20 June 2025) which provides the annotated peripheral blood smear images. All data supporting the findings of this study are contained within this openly available dataset; no new data were generated.

## Supplementary Materials

The following supporting information can be downloaded at https://www.mdpi.com/article/10.3390/curroncol33080486/s1, Figure S1:Unnormalized confusion matrix on the held-out test set. Columns correspond to ground-truth classes and rows to predicted classes. The background row and column capture missed detections (true cells predicted as background) and false positives (background predicted as a cell), respectively.
