# Supplementary material for: Deep Learning-Based Detection Model for Leukemia Cells in Peripheral Blood Smears Using YOLOv11-Large
Source: Curr Oncol. 2026 Aug 18;33(8):486. doi: 10.3390/curroncol33080486 (PMC13510383; doi:10.3390/curroncol33080486)
Supplement: Supplementary file 1 [file curroncol-33-00486-s001.zip › curroncol-4430249-supplementary.pdf]

Supplementary Materials

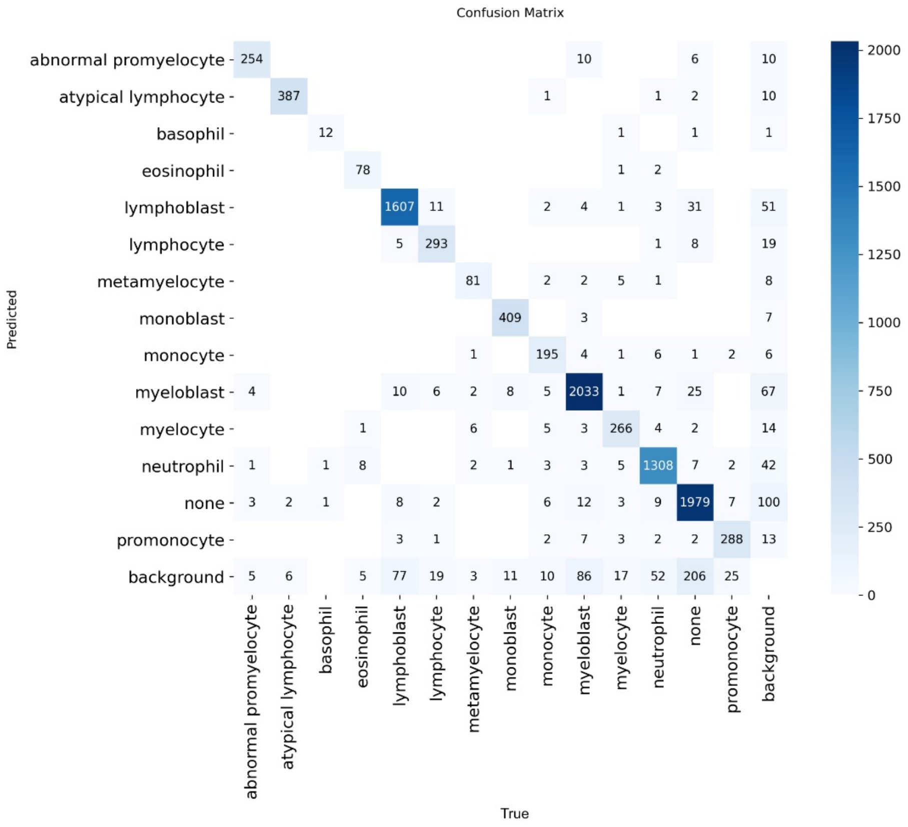

Figure S1. Unnormalized confusion matrix on the held-out test set. Columns correspond to ground-truth classes and rows to predicted classes. The background row and column capture missed detections (true cells predicted as background) and false positives (background predicted as a cell), respectively.
